# Evaluation of deep learning‐based methods for automatic detection and segmentation of brain metastases in T1‐contrast MRI for stereotactic radiosurgery

**DOI:** 10.1002/acm2.70459

**Published:** 2026-01-17

**Authors:** Zhifeng Xu, Yuqi Yang, Guanjie Wang, Yi Xue, Xinyang Zhang, Yang Dong, Liming Xu, Qi Wang, Wei Wang, Zhiyong Yuan, Sheng Huang

**Affiliations:** ^1^ National Clinical Research Center for Cancer Tianjin's Clinical Research Center for Cancer Tianjin Medical University Cancer Institute & Hospital Tianjin China; ^2^ School of Biomedical Engineering and Technology Tianjin Medical University Tianjin China

**Keywords:** brain metastases, deep‐learning based, segmentation, stereotactic radiotherapy

## Abstract

**Background:**

Brain metastases (BMs) are manually contoured during stereotactic radiosurgery (SRS) treatment planning, which is both time‐consuming and potentially inconsistent. To address these challenges, researchers have been actively developing deep learning‐based approaches for the detection and segmentation of BMs. However, a comprehensive comparative analysis of deep learning models across different frameworks remains largely absent in the current literature. This study aimed to evaluate and compare deep learning models based on different frameworks for the detection and segmentation of BMs in T1‐contrast MRI.

**Materials and Methods:**

Eight deep learning models, based on CNN, Transformer, or Mamba architectures, were trained and validated for the task of detecting and segmenting brain metastatic lesions in T1‐contrast MRI. A total of 934 patients were included, with 667 cases from publicly available datasets and 267 cases from our institution, designated for training and testing, respectively. Data were retrospectively collected and organized at our institution, and GTV defined as the total BM tumor volume delineated by the physician at the time of stereotactic radiosurgery (SRS). Additionally, labels in the publicly available dataset were modified under clinician guidance to create a BM GTV that met clinical criteria to improve ground‐truth accuracy. A BM was considered detected if the ground‐truth contour overlapped with a predicted structure. Sensitivity at both the patient‐level (proportion of patients with at least one lesion detected) and lesion‐level (proportion of ground‐truth lesions detected) were used to evaluate BM detection. Segmentation performance was assessed using several metrics: dice similarity coefficient (DSC), positive predictive value (PPV), surface DSC (sDSC), and Hausdorff distance 95% (HD95). The performance across different BM diameters was also evaluated.

**Results:**

Among the eight deep learning models, the U‐Mamba (Bot) achieved a lesion‐level sensitivity of 0.796 (95% CI: 0.779–0.812) for all sizes of BM, which was significantly higher than that of the other models, with a false positive rate of 2.46 ± 4.96 per patient. Further stratification by metastasis diameter, the sensitivity was 0.505 for BMs < 3 mm, 0.797 for BMs between 3 and 6 mm, and 0.885 for BMs between 6 and 9 mm. Moreover, U‐Mamba (Enc) demonstrated significantly higher lesion‐level segmentation performance, with DSC value of 0.632 ± 0.224. In terms of tumor boundary segmentation, nnU‐Netv2 achieved the best performance, with Surface DSC and HD95 values of 0.877 ± 0.149 and 1.770 ± 1.458 mm.

**Conclusion:**

The nnU‐Netv2 allows precise segmentation of lesion areas in T1‐contrast MRI, while U‐Mamba provide effective detection of brain metastasis, potentially aiding in treatment planning for SRS.

## 1 INTRODUCTION

Brain metastases (BMs), among the most common and devastating form of intracranial neoplasm, affect approximately 20% of adult cancer patients and are ten times more frequently than primary brain malignancies.^1^, ^2^ Traditionally, whole‐brain radiotherapy (WBRT) was considered the standard therapeutic modality for multiple brain metastases, while stereotactic radiosurgery (SRS) was primarily employed for patients with limited number of BMs, as supported by evidence from large phase III trial results.^3^, ^4^ However, some studies have highlighted the association of WBRT with long‐term declines in neurocognitive function and quality of life compared to SRS.^5^, ^6^ Furthermore, a multi‐institutional prospective study demonstrated no significant differences in overall survival, neurological function deterioration, or the need for salvage treatments between patients with 2 to 4 BMs and those with 5–10 BMs treated exclusively with SRS.^7^ Consequently, SRS has emerged as an important therapeutic modality for managing brain metastases.

In addition to the increasing evidence supporting the benefits of SRS for brain metastases, advancements in SRS techniques have also contributed to its increased utilization in the treatment of BMs. Gamma knife (GK) has historically been the leading method for intracranial SRS, but its application was constrained by the long treatment time for multiple lesions and the high demands on clinical resources for frame‐based immobilization and regulatory compliance with high‐activity Co‐60 sources.^8^ In contrast, linear accelerator (Linac)‐based SRS has emerged as a more popular and widely accessible alternative, facilitated by continuous improvements in both software and hardware that enhance stereotactic treatment capabilities. These advancements in SRS technology, combined with the lower cost and greater availability of Linac‐based systems, have further contributed to a significant increase in the proportion of patients receiving SRS, rising from 9.8% in 2004 to 25.6% in 2014.^9^

A critical step in the SRS workflow is the identification and delineation of treatment planning targets by radiation oncologists. This process, however, is time‐consuming, labor‐intensive, and subject to significant intra‐ and inter‐observer variability.^10^ Additionally, small lesions or those with low contrast relative to surrounding tissues may be overlooked. To address these challenges, there has been a growing effort to develop automated detection and segmentation techniques to improve the precision and efficiency of target contouring.

Recently, deep learning (DL)‐based auto‐segmentation methodologies have shown significant potential and robust performance in brain metastases segmentation.^11^, ^12^, ^13^, ^14^, ^15^, ^16^ Convolutional neural networks (CNNs) are particularly effective at capturing local spatial features with relatively low computational complexity. Among these, the state‐of‐the‐art convolutional neural network (CNN)‐based automatic segmentation framework, nnU‐Net (‘no‐new U‐Net’),^17^ which autonomously configures the required parameters for training, has been shown to outperform other existing approaches in international biomedical segmentation challenges using 23 public datasets. Despite these developments, researchers are exploring innovative architectures like Transformer‐based and Mamba‐based approaches for image segmentation.^18^, ^19^, ^20^, ^21^, ^22^ In contrast to CNNs, Transformers treat the images as a sequence of patches and leverage self‐attention mechanisms to capture global information, which has better capabilities to model long‐range dependencies.^23^ However, Transformers are generally very computationally expensive. More recently, Mamba‐based architectures improved state space sequence models with a selective mechanism, achieving strong capabilities in capturing both local and global information while also improving computational efficiency.^24^ Compared with ViTs, Mamba offers a favorable trade‐off by retaining strong global modeling capacity while reducing the computational overhead.

While the development of automatic artificial intelligence (AI) segmentation algorithms for brain metastases has advanced rapidly, a systematic comparison of DL models based on different frameworks remains largely absent within the research community. Moreover, while certain models have shown promising segmentation performance in general medical image tasks, their effectiveness in the context of brain metastases, particularly evaluating across extensive testing datasets, remains inadequately validated. In the present study, we thoroughly compared the performance of six different networks—nnU‐Netv2,^17^ UNETR,^25^ Swin UNETR,^18^ LightM‐UNet,^20^ LKM‐UNet,^21^ and U‐Mamba^22^—in segmenting brain metastases in T1‐weighted contrast‐enhanced magnetic resonance imaging (MRI). All aforementioned methods were implemented in the 3D nnU‐Net framework. These models were trained on publicly available datasets and further evaluated on a substantial dataset from our institution, in addition to external validation using public datasets to assess their generalizability and clinical applicability. Through this comprehensive comparative study, we aim to identify the most effective model for clinical application, offering valuable insights into the potential integration of these models into routine clinical practice for more accurate and efficient diagnostics.

## 2 METHODS

IRB approval was received from the Tianjin Medical University Cancer Institution & Hospital, and written informed consent was waived for this HIPAA compliant retrospective study.

### 2.1 Patient dataset

A total of 934 patients were included in this study. The training and validation data were obtained from publicly available datasets, comprising 562 MRI scans, with 238 scans from the 2023 BraTS‐METS brain metastasis challenge,^26^ and 324 scans from the University of California, San Francisco.^27^ Although these datasets contained multiple MRI sequences, only T1 weighted contrast‐enhanced MRI data were utilized for the purpose of this study. It should be noted that while some training cases have included patients with prior craniotomy, resection, or biopsy, these were used only in the training/validation cohort. For testing, 267 patients who has been diagnosed by clinical radiation oncologists and underwent SRS treatment between December 2020 and December 2024 at Tianjin Medical University Cancer Institution & Hospital were included, referred to as Testing Dataset 1. There were no limitations on the number or size of metastases. The primary cancer sites of these in‐house patients were: lung (170 patients, 63.7%), breast (60 patients, 22.5%), gastrointestinal (21 patients, 7.8%), kidney (eight patients, 3.0%) and other sites (eight patients, 3.0%). An additional external test set, referred to as testing dataset 2, contains 105 patients from the BrainMetsShare dataset^28^ (https://doi.org/10.71718/z66c‐qr59). All testing datasets included only non‐resected and non‐treated cases.

### 2.2 MRI acquisition parameters

Each patient at our institution underwent imaging simulation with a three‐dimensional T1‐weighted spoiled gradient‐echo MRI sequence following the administration of the contrast agent. The representative parameters from the Philips MR Ingenia Elition 3.0T simulator for the T1‐contrast images were as follows: repetition time, 4.5 ms; echo time, 2.3 ms; inversion time, 1000 ms, flip angle, 8°; matrix size, 512 × 512 × slices (length dependent); and voxel size, 0.55 × 0.55 × 1.25 $mm^{3}$.

### 2.3 Image preprocessing and brain metastases annotations

Both the training and testing MRI datasets underwent standard preprocessing steps, including conversion from DICOM to NifTI format, resampling to a uniform resolution, intensity normalization and skull stripping. Skull stripping was performed using the HD‐BET skull stripping network to eliminate the influence of non‐brain tissue on segmentation accuracy, as described in a previous study.^29^

The target segmentation in the public dataset (excluding BrainMetsShare) followed a 3‐label annotation protocol—Non‐enhancing tumor core (NETC; Label 1), Surrounding non‐enhancing FLAIR hyperintensity (SNFH; Label 2), and Enhancing Tumor (ET; Label 3).^26^ The “Tumor Core (TC) = Label 1+ Label 3” represents the gross tumor volume (GTV) used for clinical treatment in our institution. Unlike other infiltrative tumors such as gliomas, BMs typically have well‐defined borders of the contrast‐enhancing portion. Therefore, under the guidance of clinical radiation oncologists, an additional preprocessing pipeline was applied to the target regions in training dataset using Boolean operations to align with our clinical requirements. No further pre‐processing was performed prior to input into the neural network.

**FIGURE 1:**
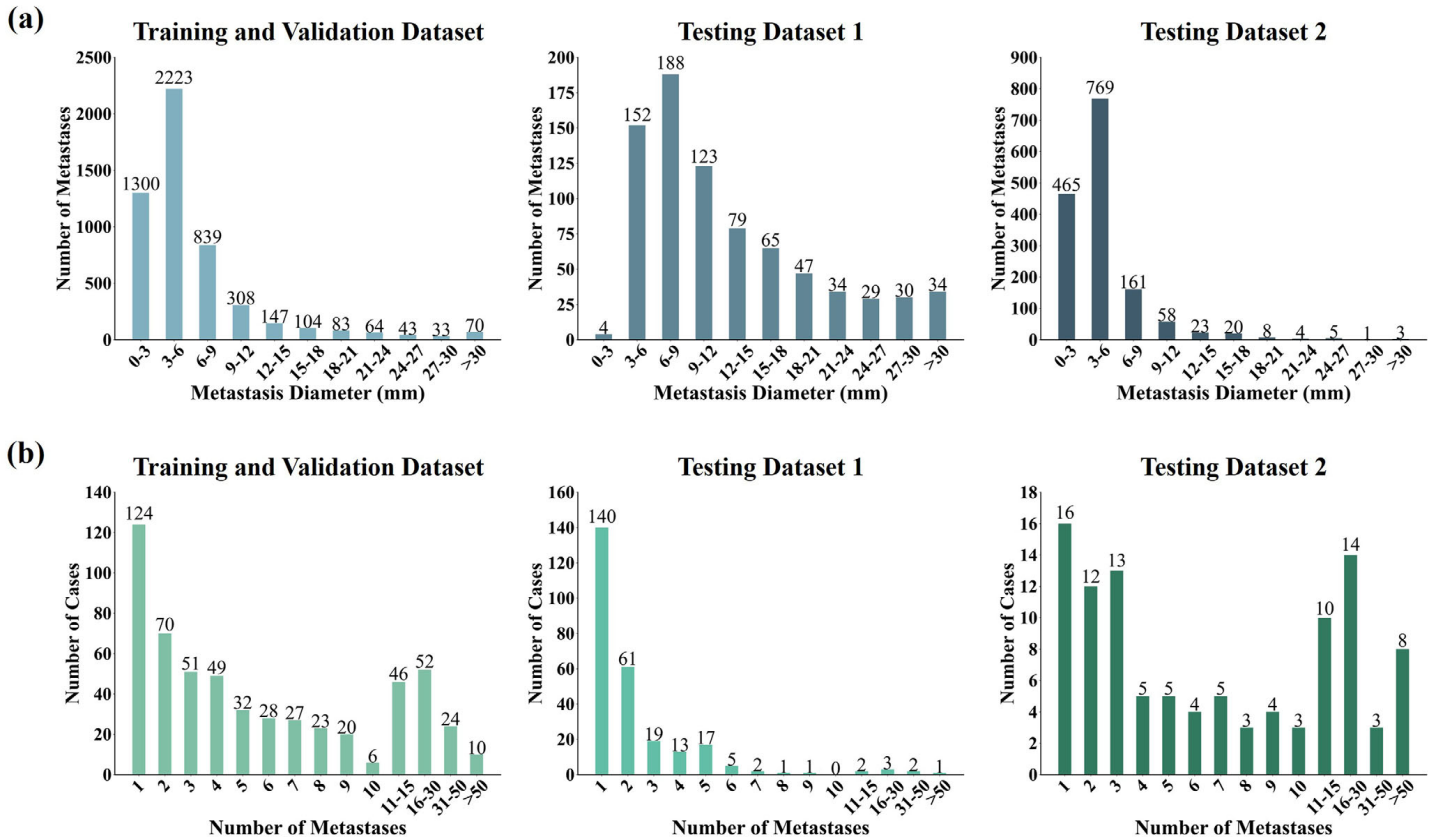
Distribution of brain metastasis diameters in the training and validation cohort, testing cohort 1, and testing cohort 2 (a) and distribution of the number of brain metastases across patients in the training and validation cohort, testing cohort 1, and testing cohort 2 (b).

The ground‐truth masks for the Testing Dataset 1 were initially contoured using the atlas method in MIM and subsequently refined by an experienced attending radiation oncologist. The identification of brain metastases and the corresponding segmentation masks were established through consensus among clinicians. The training dataset included a total of 5214 metastases, with an average of 9 BMs per patient. The testing dataset includes 2302 metastases, with 785 BMs in testing dataset 1 (an average of 3 BMs per patient) and 1517 BMs in testing dataset 2 (an average of 15 BMs per patient). Figure 1 illustrates the histogram of metastasis versus diameters(equivalent diameter, that is, the GTV considered to be a spherical) and number of lesions per patient across both the training and testing datasets, respectively.

### 2.4 Neural networks

Two prominent network architectures, convolutional neural network (CNN) and transformer, have demonstrated exceptional capabilities in biomedical image segmentation. CNN excels at capturing local features through convolution operations, making them particularly effective for pixel‐level feature extraction. However, their ability to learn long‐range dependencies is inherently limited by the constraints of the receptive fields. In contrast, Transformer provides robust long‐range modeling and achieves remarkable global context comprehension by leveraging multi‐head self‐attention mechanisms. Nevertheless, this capability comes at the expense of pixel‐level spatial modeling and entails a significant computational cost. More recently, Mamba architectures, a structure state space model (SSMs), has achieved cutting‐edge performance in handling long sequences and global contextual information through a selection mechanism and a hardware‐aware algorithm, while also enhancing computational efficiency.

To evaluate the performance of metastasis detection and segmentation, six different neural networks were implemented on our platform, including the CNN‐based network (nnU‐Netv2^17^), the Transformer‐based networks (UNETR^25^ and Swin UNETR^18^), and the Mamba‐based networks (LightM‐UNet,^20^ LKM‐UNet,^21^ and U‐Mamba^22^). nnU‐Netv2 and UNETR/Swin UNETR represent classical models of CNN and Transformer architectures, respectively, while LightM‐UNet, LKM‐UNet, and U‐Mamba are more recent models. This selection ensures a comprehensive coverage of the variability in contemporary approaches for this study.

nnU‐Netv2 follows a generic U‐Net architecture. UNETR utilizes a transformer as the U‐Net encoder, directly leveraging embedded 3D patches to effectively capture long‐range dependencies, and connects the Transformer‐based encoder to the CNN‐based decoder via skip connections. Unlike UNETR, Swin UNETR employs the Swin transformer^30^ as its encoder, combining self‐attention with a shifted windowing mechanism to extract feature representations at several resolutions. To address the challenges stemming from computational resource constraints in real clinical settings, LightM‐UNet leverages the Residual Vision Mamba Layer (RVM layer) to extract deep semantic features from images in a pure Mamba manner and model long‐range spatial dependencies. Compared to CNN and Transformer, LKM‐UNet excels in local‐neighborhood pixel‐level modeling and maintains superior in long‐range global patch‐level modeling through a novel hierarchical and bidirectional large kernel Mamba module (LM block), which composed of pixel‐level SSM (PiM) and patch‐level SSM (PaM). Finally, U‐Mamba features two variants, U‐Mamba (Bot) and U‐Mamba (Enc), which incorporate the U‐Mamba block either exclusively in the bottleneck layer or throughout all encoder blocks, respectively.

All networks were integrated into the auto‐configuration framework, utilizing the default nnU‐Net training pipeline. Among the three available configurations, the 3D U‐Net with full image resolution was employed in this study. The networks were trained for 1,000 epochs with default parameters (e.g., patch size, batch size). Additionally, the nnU‐Netv2 model underwent training with 5‐fold cross‐validation (nnU‐Netv2(5)) to further assess its impact on performance. All model training was carried out on an Ubuntu 22.04 operating system equipped with an NVIDIA GeForce RTX A6000 GPU (48 GB VRAM) and an Intel Xeon Gold 6258R CPU.

### 2.5 Statistical analysis

Detection accuracy was first evaluated using the sensitivity metric at both per‐patient and per‐metastasis levels. Additionally, the number of false positives (FPs) per patient was evaluated. Individual metastases were identified using connected‐component analysis algorithm to differentiate distinct lesions. True positive (TP) detections were defined as predicted lesions having any overlap with the corresponding ground truth mask and otherwise was considered as a false positive (FP). If any metastases of a patient were not detected, then the patient‐level result was classified as a false negative. Next, contouring accuracy was evaluated at both per‐patient and per‐metastasis levels using a broad spectrum of metrics. At the per‐patient level, we calculated the Dice Similarity Coefficient (DSC) and Positive Predictive Value (PPV). Performance at the per‐metastasis level was assessed using DSC, PPV, Surface DSC (sDSC), and Hausdorff Distance 95% (HD95). The sDSC was calculated using the same formula as volumetric DSC but applied to the segmentations surface areas rather than volumes, with a tolerance of 1 mm. Notably, sDSC has been shown to correlate strongly with the time required for contour editing.^31^

To further investigate the impact of brain metastasis size on detection accuracy and segmentation performance, tumor detection sensitivity was analyzed as a function of metastasis diameter (<3 mm, ≥3 mm to <6 mm, ≥6 mm to <9 mm, etc.). Additionally, the effect of metastases size on the DSC and sDSC was analyzed for the three models that demonstrated optimal detection performance in the different architectures. All statistical analyses were conducted utilizing GraphPad Prism software (v. 9.5.1) and Python (v. 3.11.7). Model performance was compared using the McNemar and Wilcoxon signed‐rank tests. Spearman correlation analysis was performed to assess relationships between DSC and lesion diameter. A *p*‐value of less than 0.05 was considered indicative of statistical significance.

## 3 RESULTS

### 3.1 Metastases detection

**FIGURE 2:**
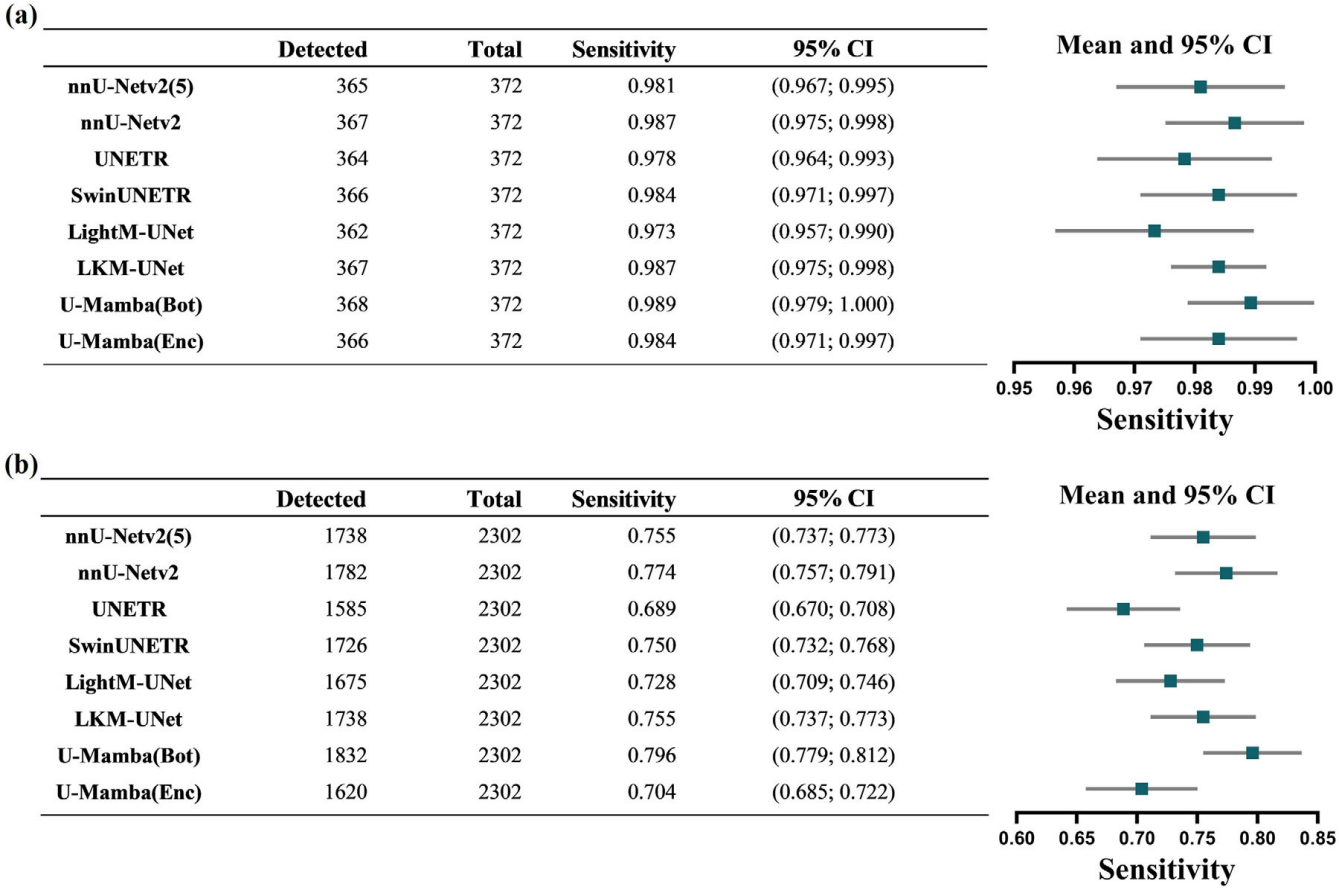
(a) Patient‐wise sensitivity analysis of brain metastasis segmentation models; (b) patient‐wise sensitivity analysis of brain metastasis segmentation models. nnU‐Netv2(5) and nnU‐Netv2 represents models trained with and without 5‐fold cross validation, respectively.

We first analyzed the detection performance of all models at both the patient level and lesion level. The patient‐level evaluation demonstrated consistently high sensitivity across all models, as shown in Figure 2a. Architecturally distinct models exhibited equivalent sensitivity performance, with no statistically significant differences observed in patient‐level sensitivity (pairwise McNemar test, *p*‐value range: 0.07–1.00).

For lesion‐level sensitivity across all metastasis sizes as shown in Figure 2b. The U‐Mamba (Bot) achieved significantly higher sensitivity of 0.796 (95% CI: 0.779–0.812) than the other models (pairwise McNemar test: *p* < 0.05), with a FP rate of 2.46 ± 4.96 per patient. The nnU‐Netv2 attained a sensitivity of 0.774 (95% CI: 0.757–0.791), slightly lower than U‐Mamba (Bot) but significantly higher than the remaining models (pairwise McNemar test: *p* < 0.05), with a FP rate of 2.03 ± 4.26 per patient. For the Transformer‐based model, UNETR demonstrated the lowest sensitivity, at 0.689 (95% CI: 0.670‐0.708), which was significantly lower than the other models (pairwise McNemar test: *p* < 0.05). Its FP rate was 3.34 ± 5.14 per patient. In addition, the lesion‐level sensitivity of nnUNetv2 (5), Swin UNETR, and LKM‐UNet was comparable, with no statistically significant differences among these models (pairwise McNemar test: *p* ≥ 0.05), and the FP rate per patient were 1.70 ± 3.51, 3.38 ± 6.43 and 1.80 ± 3.68, respectively. For LightM‐UNet and U‐Mamba (Enc), the FP rates per patient were 2.53 ± 5.47 and 2.93 ± 5.49, respectively.

**FIGURE 3:**
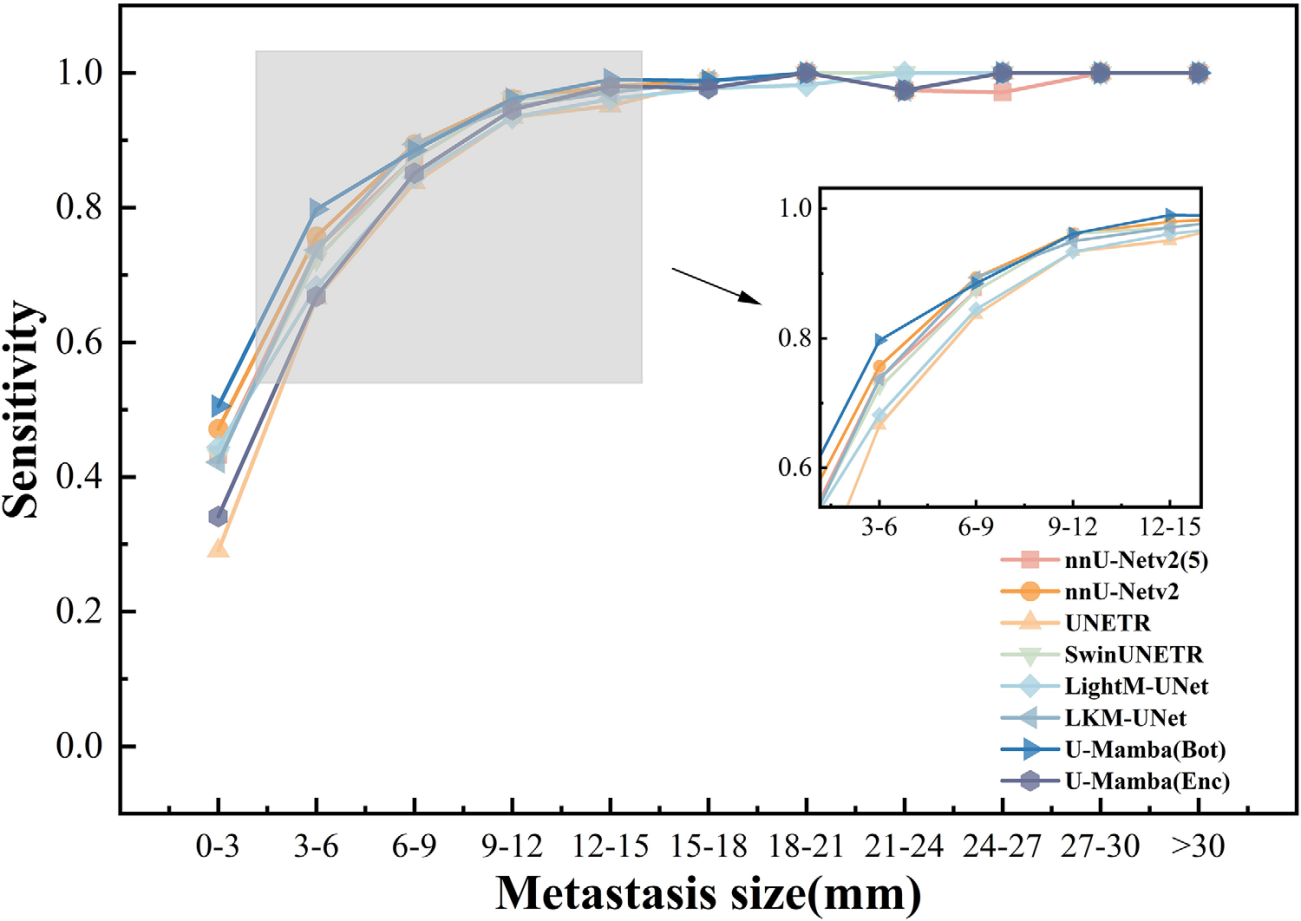
Sensitivity analysis of brain metastasis segmentation across tumor diameter ranges for various deep learning models. Overall sensitivity trends stratified by metastasis size (diameter, 0–3 mm to >30 mm).

Further stratification by metastasis diameter, as illustrated in Figure 3, demonstrated that all models exhibited robust performance for metastases larger than 9 mm in diameter (563 BMs in total), with sensitivity levels consistently above 0.93. There was no significant statistical difference between the models (pairwise McNemar test: *p* ≥ 0.05). For metastases measuring between 6 and 9 mm in diameter (349 BMs in total), most models still maintain high sensitivity. Both nnU‐Netv2 and LKM‐UNet achieved sensitivity of 0.894 (95% CI: 0.857–0.922). For metastases measuring between 3 and 6 mm in diameter (921 BMs in total), sensitivity ranged between 0.7 and 0.8 for majority of models but showed variability and a noticeable drop, particularly for UNETR and U‐Mamba (Enc), which demonstrated significant reductions in sensitivity for smaller metastases. Notably, U‐Mamba (Bot) still demonstrate exceptional robustness with a sensitivity of 0.797 (95% CI: 0.77–0.822), significantly outperforming the other models (pairwise McNemar test: *p* < 0.05). The second‐best performer was nnU‐Netv2, with a sensitivity of 0.757 (95% CI: 0.728–0.783), which was also significantly higher than the other six models (pairwise McNemar test: *p* < 0.05). In the context of more challenging smaller metastases (less than 3 mm in diameter, 469 BMs in total), U‐Mamba (Bot) consistently achieved the highest detection rate, with a sensitivity of 0.505 (95% CI: 0.46–0.55), significantly surpassing all other models (pairwise McNemar test: *p* < 0.05). For the remaining models, on the other hand, the sensitivity dropped below 0.5. Especially for UNETR, sensitivity dropped to 0.29 (95% CI: 0.251–0.333), significantly lower than the other seven models (pairwise McNemar test: *p* < 0.05). The nnU‐Netv2 achieved a sensitivity of 0.471 (95% CI: 0.426–0.516), which was significantly higher than nnU‐Netv2 (5) at 0.433 (95% CI: 0.389–0.478) (pairwise McNemar test: *p* < 0.05).

### 3.2 Metastases segmentation

We subsequently evaluated the segmentation performance of all models. Table 1 summarizes the segmentation performance metrics at both the patient‐level and lesion‐level.

**TABLE 1:** Summarized segmentation performance of deep learning models for brain metastasis on both patient‐wise and lesion‐wise. Patient‐wise metrics summarize performance per patient by comparing the union of all predicted metastases with the union of all ground‐truth metastases for that patient. Lesion‐wise metrics evaluated each individual metastasis separately. Values are reported as mean ± standard deviation. DSC: Dice similarity coefficient, PPV: Positive predictive value, sDSC: Surface DSC, HD95: 95% Hausdorff distance.

	Patient wise		Lesion wise			
Methods	DSC	PPV	DSC	PPV	sDSC	HD95 (mm)
**nnU‐Netv2(5)**	0.728 ± 0.239	0.776 ± 0.261	0.620 ± 0.230	0.752 ± 0.244	0.872 ± 0.153	1.789 ± 1.406
**nnU‐Netv2**	0.724 ± 0.243	0.765 ± 0.266	0.621 ± 0.228	0.743 ± 0.247	**0.877 ± 0.149**	**1.770 ± 1.458**
**UNETR**	0.669 ± 0.275	0.737 ± 0.286	0.602 ± 0.247	**0.773 ± 0.243**	0.829 ± 0.203	2.077 ± 1.952
**SwinUNETR**	0.721 ± 0.247	0.759 ± 0.265	0.624 ± 0.232	0.748 ± 0.246	0.871 ± 0.158	1.836 ± 1.858
**LightM‐UNet**	0.703 ± 0.253	0.748 ± 0.280	0.612 ± 0.231	0.729 ± 0.264	0.859 ± 0.166	1.917 ± 2.300
**LKM‐UNet**	**0.745 ± 0.218**	**0.794 ± 0.233**	0.616 ± 0.230	0.753 ± 0.245	0.869 ± 0.151	1.796 ± 1.423
**U‐Mamba(Bot)**	0.720 ± 0.244	0.759 ± 0.273	0.616 ± 0.224	0.715 ± 0.265	0.872 ± 0.150	1.777 ± 1.366
**U‐Mamba(Enc)**	0.699 ± 0.263	0.728 ± 0.295	**0.632 ± 0.224**	0.743 ± 0.253	0.866 ± 0.155	1.820 ± 1.469

In the patient‐level analysis, LKM‐UNet demonstrated superior performance in segmentation metrics, achieving DSC value of 0.745 ± 0.218, which were significantly higher than the other models (pairwise Wilcoxon signed rank test: *p* < 0.05) except nnU‐Netv2 (5) and nnU‐Netv2. In contrast, UNETR exhibited the lowest performance in both metrics, with DSC value of 0.669 ± 0.275, which were significantly lower than the other models (pairwise Wilcoxon signed rank test: *p* < 0.01). Additionally, LKM‐UNet achieved a significantly higher PPV of 0.794 ± 0.233 compared to the other models (pairwise Wilcoxon signed rank test: *p* < 0.05), with the exception of nnU‐Netv2 (5).

In the per‐metastasis level, U‐Mamba (Enc) demonstrated superior segmentation performance compared to other models in the DSC evaluation metrics, achieving a DSC of 0.632 ± 0.224 (pairwise Wilcoxon signed rank test: *p* < 0.05). UNETR achieved the highest PPV of 0.889 ± 0.226 (pairwise Wilcoxon signed rank test: *p* < 0.05). In evaluating the accuracy of edge delineation for the target lesions, nnU‐Netv2 outperformed all other models, achieving sDSC and HD95 values of 0.877 ± 0.149 and 1.770 ± 1.458 mm, with statistically significant differences (pairwise Wilcoxon signed rank test: *p* < 0.05).

**FIGURE 4:**
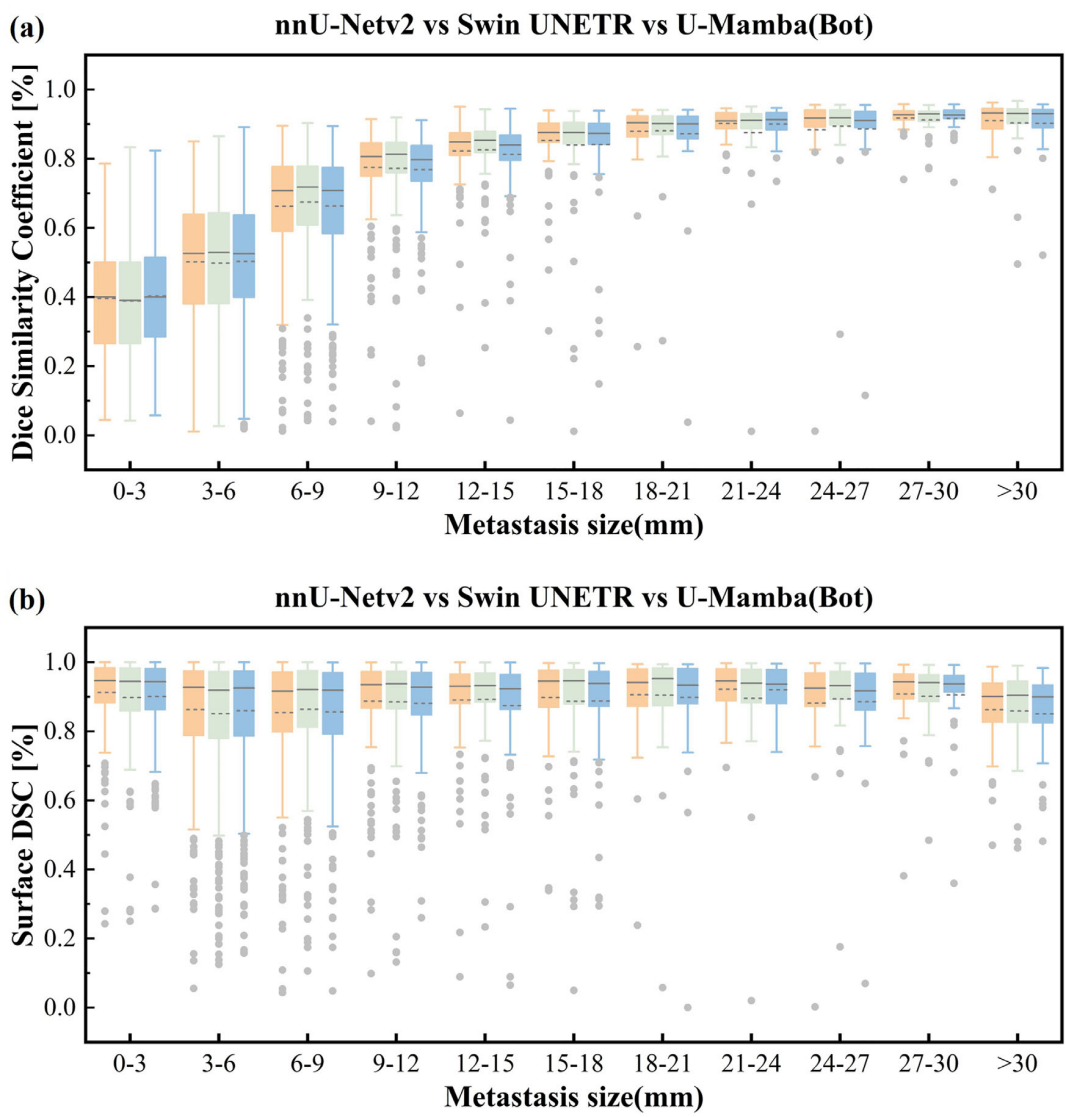
Performance comparison of BMs segmentation across tumor diameter ranges using nnU‐Netv2 (orange), Swin UNETR (green), and U‐Mamba (Bot) (blue). (a) Volumetric segmentation accuracy measured by DSC, grouped by metastasis diameter (0–3 mm to >30 mm); (b) boundary segmentation precision evaluated through surface DSC for the same diameter intervals.

Further stratification by metastasis diameter for segmentation accuracy evaluation is present in Figure 4. We selected three models that demonstrated optimal detection performance across different architectures—nnU‐Netv2, Swin UNETR, and U‐Mamba (Bot). For metastases larger than 12 mm in diameter, all three models achieved DSC values exceeding 0.75. For metastases between 6 and 9 mm in diameter, Swin UNETR achieved significantly higher DSC value of 0.674 compared to the other two models (pairwise Wilcoxon signed rank test: *p* < 0.05). Segmentation performances stratified for smaller BMs (0‐3 mm and 3–6 mm), overall DSC values decreased to 0.388–0.403 and 0.468–0.503, respectively. Notably, U‐Mamba (Bot) achieved significantly higher DSC values within these ranges. A significant positive correlation was observed between DSC and metastasis diameter (Spearman's rank correlation coefficient, *p* < 0.01). Additionally, sDSC values for all three models remained above 0.85, with no significant correlation with metastasis diameter.

**FIGURE 5:**
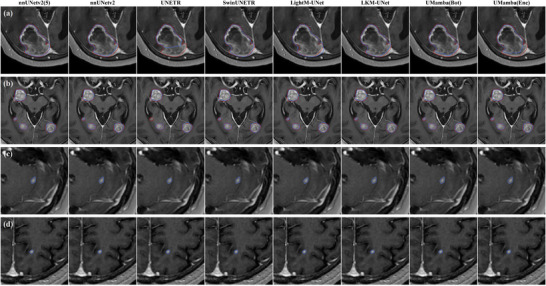
Example detection and segmentation predictions for 4 representative patients. The physician metastasis GTV contours are shaded in red line and the model predictions are outlined in blue line. (a) Is a case of solitary BM, (b) represents a case of multiple BMs, (c) illustrates a false positive example, likely in the dural region, and (d) presents an example of a lesion missed by clinical radiation oncologists.

Representative cases of the ground truth and DL segmentation masks are shown in Figure 5.

## 4 DISCUSSION

In this study, we systematically evaluated the performance of various state‐of‐the‐art deep learning networks for brain metastases detection and segmentation in T1‐contrast MRI, with a particular focus on the impact of metastasis size and the challenges associated with detecting smaller lesions. Our analysis was conducted on an extensive test set, consisting of 372 patients and 2302 discrete brain metastases, sourced from both our institution and publicly available dataset. This dataset encompassed a diverse range of lesion sizes, intracranial locations, and primary cancer types, providing a robust foundation for our evaluation. Concurrently, the study was designed such that the DL models were trained exclusively on publicly available datasets derived from multiple sources, ensuring that the models' generalizability to perform consistently across varied patient populations were reflected.

Our findings indicate that CNN‐based nnU‐Netv2 achieves superior BMs segmentation accuracy at lesion boundaries, benefiting from its strong inductive biases for local information and self‐configuring pipeline. The recently proposed Mamba architecture alse exhibits significant advantages in segmentation tasks, with U‐Mamba (Enc) outperforming all other models in DSC. Furthermore, with the integration of SSMs, the Mamba‐based U‐Mamba (Bot) further enhances BM detection accuracy, achieving a lesion‐level sensitivity of 0.796 (95% CI: 0.779–0.812)—significantly surpassing CNN‐ and Transformer‐based models. This highlights that an encoder combining the local feature extraction capabilities of CNN with the long‐range dependency modeling of SSMs provides a more practical and effective solution to overcome detection limitations. Notably, although Transformers posses strong representational capacity, they are relatively data‐hungry and typically benefit from large‐scale pretraining.^32^ In our study, all Transformer models are trained from scratch on small‐scale datasets, potentially leading to the observed difficulties in out‐competing other models.

Interestingly, when assessing the impact of 5‐fold cross‐validation on model performance, we found slight discrepancies between our findings and a previous study that suggested 5‐fold cross‐validation improves the reliability of results.^33^ For instance, nnU‐Netv2 trained on the full dataset demonstrates marginally higher lesion‐level sensitivity than its 5‐fold cross‐validation counterpart (nnU‐Netv2 (5)). Additionally, no statistically significant differences were observed between the two models in patient‐level DSC metrics (pairwise Wilcoxon signed‐rank test, *p* > 0.05). One potiential explanation for this discrepancy is the inability of the model to adequately learn the data distribution due to the limitations in the training dataset, resulting in overly optimistic estimates of its generalization ability.^34^ As a result, in scenarios involving constrained datasets or complex feature spaces, 5‐fold cross‐validation should not be universally assumed to be superior to alternative approaches (e.g., full training set validation). Specific parameter configurations or inherent data characteristics may render other methodologies more advantageous under such conditions.

Recent studies on developing and testing deep learning‐based models to automatically detect and segment BMs on unimodal or multimodal MRI (CT+MRI) has been growing rapidly.^11^, ^35^, ^36^, ^37^, ^38^ Ziyaee et al. utilizing 1051 patients to train an adaptive nnU‐Net model also showed a stratified performance of BM size, with detection sensitivities of 0.683, 0.698, 0.958, and 0.849, and segmented DSCs of 0.307, 0.710, 0.865 and 0.804 for BMs < 3 mm, 3–6 mm, > 6 mm, and across all sizes, respectively^35^ Additionally, Zhou et al. create a two‐stage DL networks (MetNet) using post‐contrast 3D T1‐weighted spoiled gradient echo MRIs from 768 cases, which achieved an overall BM detection sensitivity of 0.85 while maintaining a low FP rate. Stratified by lesion size, MetNet demonstrated sensitivities of 0.25, 0.87, and 0.99 for BMs < 3 mm, 3–6 mm, >6 mm, respectively, validated on 186 test cases^36^ We compared the models we trained with the aforementioned published findings and found that most of our models performed comparably to or even better than the models trained using multimodal images. Among them, the U‐Mamba (Bot) model achieved detection sensitivities of 0.505, 0.797, and 0.944 for BMs < 3 mm, 3–6 mm, and > 6 mm, respectively, demonstrating competitive performance against existing approaches. For multimodal research, Hsu et al. demonstrated that the multimodal model (T1+C and CECT) achieved comparable overall detection sensitivity to the single‐modality model (T1+C), but significantly outperformed it in reducing false positives and improving segmentation quality.^11^ Buchner et al. reported that a T1‐CE is sufficient to achieve high‐quality detection and segmentation of brain metastases, while multimodal inputs (such as the addition of T1 or T2‐FLAIR) did not provide significant benefits and may even increase the number of false positives.^38^

This DL‐based automatic segmentation method can assist radiation oncologists in accurately detecting and precisely delineating multiple intracranial brain metastases, thereby improving the efficiency and effectively supporting the development of personalized treatment plans. Due to the characteristics of brain metastases, such as multiplicity, dispersion, and miniaturization, this method can effectively compensate for the shortcomings of omitting tumor regions due to the above mentioned reasons or the varying cognitive level of clinicians. For example, as shown in Figure 5D, DL‐based methods successfully detected a small lesion that was initially overlooked by the clinician, highlighting the potential of DL to reduce the risk of small lesions being overlooked. In addition, DL provides a standardized and reproducible contouring process to reduce intra‐observer variablility and mitigate inter‐observer variability through high‐quality data. However, it is important to note that because our training set was sourced exclusively from public datasets, the images generally have lower spatial resolution. As a result, when models trained on these data are evaluated on clinical images, their performance in detecting small tumors may be suboptimal. This is consistent with our findings: even U‐Mamba (Bot) achieved a detection rate of only 0.505 for brain metastases < 3 mm in diameter. This limitation may also increase the likelihood of misdiagnosis—for example, misclassifying the dura mater, surgical healing, or tiny blood vessels as tumors. Therefore, constructing a data‐driven BMs detection and segmentation network based on high‐quality BMs datasets, so as to further enhance the detection and segmentation performance for small lesions, and subjecting it to iterative validation in clinical settings, should be a key focus of future research.

Our study has several limitations. First, apart from publicly available datasets, the remaining data used in this study were sourced from the same institution, without considering the variability in equipment and scanning parameters across different institutions. To further validate the generalizability of the model, it is necessary to include multi‐center patient data for evaluation. Second, different skull‐stripping methods can affect segmentation performance. The impact of skull stripping by HD‐BET was not analyzed in our study. Finally, volumetric evaluation metrics are highly sensitive to changes for small volume target. For instance, one voxel (0.5 mm × 0.5 mm × 1 mm) deviation in 3 mm brain metastases may lead to a 6% decline in the DSC. Although we supplemented our analysis with boundary evaluation metrics sDSC, we have not yet assessed which metrics have a substantive impact on clinical decision‐making, such as the time efficiency and clinical effectiveness of target segmentation. One consideration for future work is to time the additional manual editting and futher assess the edited contour with ground truth. This would further quantify the acceptability of the AI‐generated segmentations.

## 5 CONCLUSIONS

This work reports a comprehensive study on various deep learning models for brain metastasis detection and segmentation on 3D T1‐weight contrast‐enhanced MRI, covering different metastasis sizes for different primary cancer types and the extent of metastases per patient. Although the classic CNN architecture nnU‐Netv2—designed with an adaptive mechanism—demonstrates exceptionally high performance in delineating the edges of brain metastases, our findings indicate that U‐Mamba, based on the novel Mamba architecture, can further enhance the detection and segmentation performance. In the future, further research is required to improve the detection and segmentation performance, especially for small lesions, by modifying certain hyperparameters and adding functional features, ultimately aiming to enhance their clinical applicability and patient outcome.

## AUTHOR CONTRIBUTIONS

Zhifeng Xu: Performed data collection and experiments, analyzed the results, and wrote the manuscript. Yuqi Yang: Provided guidance for the experiments and manuscript writing. Guanjie Wang: Assisted in data collection. Sheng Huang and Yi Xue: provided funding support for the research. Zhiyong Yuan and Sheng Huang: Supervised the project, provided conceptual input, and approved the final version of the manuscript. All authors participated in the revision of the manuscript.

## CONFLICT OF INTEREST STATEMENT

The authors declare that the research was conducted in the absence of any commercial or financial relationships that could be construed as a potential conflict of interest.
